# Some Thoughts, in Lighter Vein, on the Practice of Medicine from a Business Standpoint1Address by the retiring president at the meeting of the Alumni Association of the University of Buffalo, April 26, 1901.

**Published:** 1901-08

**Authors:** Devillo W. Harrington

**Affiliations:** Buffalo, N. Y.


### Some Thoughts, in Lighter Vein, on the Practice of Medicine From a Business Standpoint.[1]

By DEVILLO W. HARRINGTON, M. D., Buffalo, N. Y.

IT OCCURRED to me, a few days since, that it was customary for the President of the Alumni Association, to deliver an address upon some medical topic, at the expiration of his term of office. I have listened to these addresses for nearly thirty years. They have been, without exception, both interesting and instructive. I find it particularly difficult to choose a subject that has not already been written up by those better able and with more time than I find at my disposal.

Our Alumni anniversaries are not so much to listen to dry papers upon medical topics, as to demonstrate our loyalty to our alma mater and to renew the friendships made when we were students.

I have chosen for the subject of my talk to you, Some thoughts in lighter vein on the practice of medicine from a business standpoint, or as Frank Lydston would say, "the commercial aspect of physics." I promise to take but a few moments of your time, and not to load you up with statistics, but simply to give a few ideas and facts that I have learned during the thirty years of my practice in this city.

Few doctors at the beginning of their professional career have any idea of business methods, most of them lack even a knowledge of rudimentary book-keeping. They are proverbially, "poor financiers." Few of them, upon reaching the age of sixty-four—the age at which this beneficent republic has decided that a man has outlived his usefulness—have accumulated enough to retire from practice. The amount accumulated often bears no relationship to the amount of work done. This is more often due to bad financiering, loose business methods, bad investments, puerile methods of book-keeping and making collections.

The iron clad rates for professional work, which emanated from our county societies, and of which the present generation of physicians knows very little, was a mistake from its very inception. Charges should be made in proportion to a man's means and income. It is inconsistent to charge a man whose income is $10.00 a week, the same fees that you would charge a millionaire. By this I do not mean but that you are to devote just

1. Addr ess by the retiring president at the meeting of the Alumni Association of the University of Buffalo, April 26, 1901.

as much time, and take just as good care of the poor, even those who are not able to pay anything, as of those who are able to pay large fees.

It is, perhaps, not necessary to warn doctors of the risks of investing their money in stock companies or syndicates, that offer as inducements large rates of interest. Do not be deluded into signing notes, even to accommodate patients. Be able to say "no" with a capital "N." Do not go on any one's bond, even when a representation is made to you that it is only a form and that you will not be liable. The last bond of this kind I signed was for a doctor; he was a curator in this college for many years. It cost me $2,500. He is dead now and I hope and trust that he received sentence from a higher court. Live within your means, pay your bill, promptly, as you expect others to pay you.

A blue book was published in this city a few years ago, in which was given a list of those who did not pay for medical attendance. This work was enlarged in later years, until it included all classes of debtors, and I am sorry to say, that the number of doctors names, in this book of delinquents, outnumbered those in almost any other line of business.

Do not allow your office to be a lounging place, do not get on familiar terms with druggists. It is sometimes necessary to talk plainly to a druggist whom you have found substituting or dealing out your prescriptions to his friends. Do not get chummy with your patients. I was in a doctor's office, not long since, when a young man was ushered in, who said to the office boy, "Is the Doc. in?" "No," answered the boy, "but the doctor is." Make short calls. Long professional calls tend to gossip and revealing professional secrets, which is the most contemptible thing a doctor can do.

Most of you will remember the case of Dr. Playfair, who amused his wife by giving her the details in a case he had attended; she was indiscreet in repeating it and it cost him $80,000 in the suit for slander which followed. He claimed this was due to the lack of fairplay on the part of some of his fellow practitioners.

It is the duty of every physician to recommend a patient to a specialist for any particular ailment, when he realises that they can receive better care than he is able to give them. It is the duty of a physician to consent to a consultation whenever the friends of the patient desire it. The honest physician never loses anything by asking for a consultation. Making contracts with lodges and fraternal associations, for doing professional work for a stated sum should be discouraged. There is a great tendency to this line of work among the younger members of the profession.

Be honest with your patients. Be cheerful in the sick room without levity. Too many men, whom the Creator intended for undertakers, unfortunately drift into the medical profession. Do not give advice in family matters, particularly in settling differences which doctors sometimes see.

The young doctor who marries, hoping and expecting that it is going to increase his practice will be sadly disappointed. Mark Twain's advice to young men, to marry young and if necessary often does not apply to doctors. It seems wiser for a doctor to postpone his matrimonial venture until he has a home and sufficient income to support a wife in the way she has been accustomed to live.

The practice of attending ministers and their families without charge, seems hardly just; they are often better able to pay than very many of their parishioners. I was attending a minister a few weeks since when he informed me that he had prayed for me every night for more than twenty years; upon inquiry as to this especial act of kindness, he informed me that he prayed for all sinners. Do not connect yourself with the church simply for the purpose of getting business. Philip Brooks once said, that it took a brave man and an honest one to be a sceptic, but that any coward could be a hypocrite.

Always speak well, if at all, of your fellow practitioners. It is not wise to discuss or criticise homeopathy, osteopathy or the doctrines of christian science. These are the isms that thrive under prosecution. We all remember the beautiful old hymn, "Salvation is free, to you and me," and yet it costs more in this country to save souls than to save life and relieve suffering.

There is another and a brighter side to this picture of seemingly financial preferment. The Goddess of Justice, though fickle and capricious at times, is no respector of persons, which accounts for the fact that there are more ministers in state prisons and penitentiaries than there are doctors. The young man who expects to get business by devoting a large part of his time to society is going to be sadly disappointed. Do not forget your office hours. Dr. White used to say, "take good care of your office hours and your office hours will take care of you." Take a vacation each year. A man can do better and more satisfactory work who takes a proper amount of recreation. Dress properly and becomingly. In my younger days, I remember a doctor in this city, who was one of the consulting physicians of the Buffalo General Hospital, who always attended his office hours in his shirt sleeves. I met a young doctor last fall who was wearing a silk hat, bobtail coat and yellow shoes and who seemed to think he was the pink of perfection. Do not smoke during working hours. Sick people are particularly sensitive to bad odors, and it is to be hoped that no doctor will visit the sick-room smelling like a cigar factory. Do not confine your reading entirely to medical books. Speaking of medical books, it would be better for the profession and the world at large, if every medical book, written twenty years ago or more, could be burned.

Be considerate to the younger members of the profession. Remember that you were young once yourself. I remember being called some years ago, in consultation with one of the older members of the profession, who refused to meet me, giving as his reason, that he never consulted with a man younger than himself. I consider that I have been the gainer by having young men associated with me for the past twenty years. They have had opportunities for study in many branches of which we knew but little in my student days. Our knowledge of microscopy, at that time, consisted principally in being able to discover the last remittance from home and our knowledge of chemistry was little more than being able to mix drinks.

Patients are often ungrateful; those that we do the most for usually show the least gratitude and are the ones who change doctors without any excuse or explanation. In our dealing with patients, we soon learn that there is a limit to genius and ability, but there is no limit to human stupidity.

A doctor is expected to elevate and educate the masses to the proper understanding of the rules and regulations of health and hygiene, to teach, both by precept and example, temperance, honesty, charity and morality.

By following these rules it is to be hoped that the recording angel may drop a tear and blot out any short comings for each and every one of the 140,000 physicians in this country.

1430 MAIN STREET.

